# Interventional Stroke Care in the Era of COVID-19

**DOI:** 10.3389/fneur.2020.00468

**Published:** 2020-05-08

**Authors:** Hisham Salahuddin, Alicia C. Castonguay, Syed F. Zaidi, Richard Burgess, Ashutosh P. Jadhav, Mouhammad A. Jumaa

**Affiliations:** ^1^Department of Neurology, University of Toledo, Toledo, OH, United States; ^2^Promedica Neurosciences Center, Toledo, OH, United States; ^3^Department of Neurology, University of Pittsburgh Medical Center, Pittsburgh, PA, United States

**Keywords:** COVID-19, stroke, pandemic, thrombectomy, ischemic, coronavirus, cerebrovascular, neurointervention

## Abstract

The current coronavirus disease (COVID-19) pandemic caused by severe acute respiratory syndrome coronavirus 2 has led to immense strain on healthcare systems and workers. Patients with severe symptoms of COVID-19 may also present with acute neurological emergencies such as ischemic stroke. Ischemic stroke in these patients may result from COVID-19 related complications or decompensation of previously asymptomatic cerebrovascular disorders, or concurrent ischemic stroke from common stroke risk factors in a patient with COVID-19. Acute ischemic stroke patients with large vessel occlusions require emergent triage, intensive care, and mechanical thrombectomy. Management of patients with large vessel occlusions (LVO) requires special considerations in the current pandemic. Physicians must now account for prognosis of severe COVID-19, resource utilization, and risk of infection to healthcare workers when determining eligibility for mechanical thrombectomy (MT). Here, we describe important prognostic factors including age, laboratory, and imaging findings to consider for MT selection and provide suggestions for taking care of patients with LVO and possible or confirmed COVID-19. It is recommended to perform MT in patients within the established guidelines, and consider a conservative approach in cases where there is clinical equipoise to minimize futile reperfusion. Lastly, we describe an illustrative case of a patient with ischemic stroke and COVID-19.

## Introduction

The novel severe acute respiratory syndrome coronavirus 2 (SARS-CoV-2), the causative agent of coronavirus disease 2019 (COVID-19), was first identified in Wuhan, China (1). High rates of transmissibility, primarily through airborne droplets and aerosols and international travel, have led to a worldwide pandemic. The United States (US) leads the world in the number of COVID-19 cases, and there are growing concerns regarding the potential strain on its healthcare systems due to the intensive care needed for critically ill COVID-19 patients (2). Initial reports from other countries have already highlighted the stress of COVID-19 on their intensive care units (ICU) and resources (3). A recent case series of critically ill COVID-19 patients in Seattle, Washington reported a median ICU stay of 14 days and a median duration of mechanical ventilation of 10 days (4). With emergency departments and ICUs triaging and caring for increasing numbers of COVID-19 patients, there is little doubt that the COVID-19 pandemic will have a tremendous impact on available resources for the triage and treatment of acute ischemic stroke (AIS). Here, we present an overview of COVID-19, recommendations for acute stroke care and treatment, and provide an illustrative case study of stroke in a COVID-19 positive patient.

## COVID-19

### Incidence and Mortality

Overall mortality rates in COVID-19 are dependent on gender, age, and underlying co-morbidities (5). Males are more likely to have severe symptoms and have a higher rate of mortality than females (6, 7). In a study of 44,672 confirmed COVID-19 patients in China, 80% of those who died were 65 years or older (8). Patients between 60 and 69, 70 and 79, and above 80 years of age have a mortality rate of ~4, 10, and 21%, respectively (5, 9). Patients with severe COVID-19 admitted to ICU have mortality rates of ~26% (7). Furthermore, patients with co-morbidities are considered high-risk, specifically those with hypertension (10). Of 1,625 deaths in Italy, 25% of patients had one co-morbidity, 25% had two, almost half had three or more co-morbidities, and <1% of patients did not have any co-morbidities (5). Shared risk factors for stroke and severe COVID-19 include diabetes mellitus, hypertension, coronary artery disease, and chronic kidney disease (11–13). In a Chinese study of 191 patients, hypertension (OR 3.05; 1.57–5.92), diabetes (OR 2.85; 1.35–6.05), and coronary artery disease (OR 21.4; 4.6–98.7) were independently associated with death from COVID-19 (10). Another study revealed that those with cardiovascular disease and an elevated troponin had a mortality rate of 69% (14).

Early studies of COVID-19 show that multiple factors contribute to high rates of mortality in selected populations. Patients from extended care facilities may have rates of mortality as high as 34% (15). In addition to considerations of age, co-morbidities, lab, and imaging findings, the MuLBSTA scoring system for viral pneumonia has been shown to be an effective predictor of 90-day mortality. Presence of multilobe infiltrates, lymphopenia (≤ 0.8 × 10^9^/L), bacterial co-infection, smoking status, age, and hypertension are scored to give estimated mortality ranges from 2% to >69% (16).

### Presentation

#### Symptoms

Mean time from contact exposure to development of COVID-19 symptoms is ~5 days, although a history of contact exposure or travel is not always present (17). The rate of asymptomatic carriers is currently unknown, but preliminary estimates range from 30 to 80% (18, 19). Of those who develop clinical symptoms, 80% experience a mild clinical course (20). Presenting symptoms may include fever, cough, myalgia, and/or fatigue, and less commonly nausea or vomiting, sputum production, and diarrhea (21, 22). In one study, median time from onset of symptoms to hospitalization was 7 days, to development of dyspnea was 8 days, and to severe respiratory distress was 9 days (23). Patients with severe COVID-19 may develop severe respiratory distress, acute kidney injury, sepsis, secondary infection, coagulopathy, or acute cardiac injury at presentation or within a few days of admission (10). Patients who have longer times from symptom onset to hospitalization or present with respiratory distress are at higher risk of developing severe COVID-19 (24).

The WHO defines mild COVID-19 as patients with uncomplicated upper respiratory tract infections often with non-specific symptoms such as fever, cough, dyspnea, diarrhea, nausea, or vomiting. Severe COVID-19 is usually characterized by severe pneumonia (respiratory rate >30 /min, severe respiratory distress, or oxygen saturation ≤93%), acute respiratory distress syndrome, sepsis, or septic shock (25).

#### Laboratory and Imaging Findings

Although rapid bedside testing for COVID-19 is now available, multiple laboratory and imaging markers can help determine severe cases of COVID-19. Low white blood counts (WBC), low lymphocyte-to-WBC ratio, elevated bilirubin, C-Reactive Protein (CRP), D-dimer, and lactate dehydrogenase (LDH) may be markers in patients who develop severe disease (21, 22, 24, 26). Computed tomography (CT) chest imaging often reveals characteristic findings such as ground-glass opacities distributed in the lung periphery and patchy shadowing bilaterally, which can aid in determining the extent and severity of disease (27).

#### Neurological Findings

Initial reports from Wuhan, China revealed that ~36.4% of severe COVID-19 patients develop neurological symptoms including impaired level of consciousness, headache, dizziness, seizures, dysgeusia, anosmia, dysphagia, and muscle pain (28, 29). Although patients with neurological symptoms are more likely to have severe COVID-19 disease, many present with neurological symptoms as the only presenting symptom of COVID-19. Anosmia is an early, common, specific, and often initial symptom of COVID-19, which may improve with resolution of illness (30). Anosmia and dysgeusia have been reported in over 85% of patients with infection (31). CNS involvement has been hypothesized to occur from hematogenous spread or direct involvement of the olfactory bulb and retrograde neuronal invasion (32, 33). Case reports of acute hemorrhagic necrotizing encephalopathy, meningoencephalitis, Miller Fisher syndrome, Guillain-Barre syndrome, and acute myelitis in COVID-19 patients have recently been described (29, 34–37). COVID-19 has also been detected in CSF and long term neurological sequelae of COVID-19 remain unknown (38).

#### Stroke

Ischemic stroke has been reported to occur in ~5% of COVID-19 patients admitted to hospital, which included patients with large vessel stenosis, small vessel disease, and cardioembolic strokes (39). Recent reports have emerged of patients younger than 50 years with LVO with minimal or no symptoms of COVID-19 (40). However, most patients with stroke (ischemic stroke, cerebral venous thrombosis, and intracranial hemorrhage) are more likely to be older and present with severe COVID-19 symptoms (39). Mortality rates of patients with severe COVID-19 and a history of cerebrovascular disease range from 20% (6 of 30) to 100% (7 of 7) (6, 12). Multiple risk factors for severe COVID-19 have been identified, but cerebrovascular disease has been shown to be a negative prognostic factor in those with COVID-19 (39).

Numerous theories have emerged regarding the pathogenesis of severe disease in patients with COVID-19. For patients with stroke, a proinflammatory state triggered by cytokines may contribute to vascular endothelial injury or a hypercoagulable state which may result in extensive thrombosis and microvascular dysfunction (41–43). These hypotheses are supported by elevated D-dimers in patients with severe COVID-19, anecdotal reports of patients with ST-elevation myocardial infarction (STEMI) and normal coronary arteries (44), and reports of limb ischemia (43, 45). Furthermore, use of heparin has been shown to reduce mortality in patients with severe COVID-19 (46). Lung biopsy of a patient with COVID-19 has shown small vessel hyperplasia, vessel wall thickening, luminal stenosis, occlusion, and micro-thrombosis (47).

Ischemic stroke and COVID-19 may occur concurrently either from common stroke pathologies such as atrial fibrillation in patients with mild or asymptomatic COVID-19, as a complication of severe COVID-19 pathology such as hypercoagulability or a proinflammatory state, or due to worsening hypoxia and decompensation in patients with previously asymptomatic cerebrovascular disorders such as carotid occlusion or moyamoya disease/syndrome.

### Acute Stroke Care Considerations

Acute stroke care continues to be a vital part of emergency care, even amid a pandemic or crisis. The American Heart Association/American Stroke Association (AHA/ASA) has provided temporary emergency guidance for stroke centers during the current crisis which includes admission of stable stroke patients directly to step down units instead of the ICU and encourages the use of telemedicine for acute stroke patients (48). Acute ischemic stroke (AIS) patients with large vessel occlusions (LVO) benefit from mechanical thrombectomy (MT) within 24 h from “last seen normal” time (49–55). Although the use of intravenous tPA for AIS has not been studied in patients with COVID-19, its use is imperative for eligible patients with AIS. Given the current crisis, selection of patients for MT poses new challenges and requires refinement of established protocols. Recently published recommendations from the Society of NeuroInterventional Surgery (SNIS) recommend that MT should be performed in patients in which there is high-quality data to support benefit (56). The decision to perform MT in patients outside established guidelines should be pursued in the context of resource availability and balanced against the risks and benefits to the patient as well as the treating health-care providers.

Drastic changes have occurred during the current COVID-19 crisis. Treatment goals have evolved from providing the best clinical care for patients to providing the best clinical care for patients without overutilization of resources or compromising healthcare worker safety. Treatment of life-threatening, time urgent conditions such as AIS and STEMI have always focused on providing the correct treatment for those who may benefit as early as possible. The concern of contact with possible COVID-19 positive patients may result in delayed treatment and triage, which has already been reported in China as door-to-device times for STEMI have increased by 25 min (57). Treatment of LVO will likely see similar increases in times as regional, hospital bypass, and mobile stroke unit protocols change, and healthcare workers don and doff personal protective equipment (PPE).

MT in patients older than 80 years of age has not been extensively evaluated in randomized clinical studies. In the HERMES meta-analysis, patients over 80 years had a mortality rate of 15.3% and a good clinical outcome in 29.8% (58). Real-world experience of patients undergoing MT over 80 years of age revealed a good functional outcome in 21% and a higher rate of intracranial hemorrhage than younger patients (59). Mortality in patients over 80 is significantly higher than younger patients (33 vs. 14%) (60). Patients over 80 years of age also have high rates of futile reperfusion (50%) (61). Although rates of mortality are higher in older patients, there remains a benefit in terms of functional clinical outcome in patients without COVID-19. The high rate of mortality of COVID-19 in this age group warrants careful evaluation of risks and benefits when mechanical thrombectomy is considered.

### Screening and Pre-hospital Care

Early suspicion of COVID-19 in patients is vital for stroke team preparation. Screening for fever, contact with COVID + patients, and recent cough should ideally be performed by 911 dispatchers and EMT should be informed prior to on-scene arrival. For inpatient stroke codes, clinical information can be obtained from the patient's chart and nursing staff prior to in-person assessment. Telemedicine can be utilized both in the emergency department as well as for inpatient stroke codes to minimize provider contact with potential COVID-19 patients. Details of providing hyperacute stroke code care have been recently published (62). Urgent lab draws and rapid bedside testing for COVID-19 should be performed on arrival or during an inpatient code stroke, and if COVID-19 is suspected or clinical history cannot be obtained accurately, a CT chest may be performed prior to transport to the neuroangiography suite (NAS) (63).

### Patient Selection

Prior to selecting patients for MT, risk factors for severe disease and mortality from COVID-19 and potential risks to healthcare workers need to be thoroughly evaluated. Patients for whom there is clinical equipoise, such as those with anterior or posterior circulation LVO and low NIHSS (National Institute of Health Stroke Scale), distal occlusions, low ASPECTS (Alberta Stroke Program Early CT Score), or minimal to moderate penumbral tissue, should be managed conservatively. Patients with an ASPECTS score of 5 or less have poor functional outcomes, high rates of futile reperfusion, and should be managed conservatively in the current climate (64, 65). Patients with low NIHSS and LVO, should be monitored closely, given possible subsequent clinical deterioration from COVID-19 related hypoxia or altered hemodynamics. In cases of subsequent clinical deterioration, MT should be performed if they meet established LVO guidelines. Institutions enrolling patients with clinical equipoise for MT in randomized clinical trials should halt enrollment and provide medical management in these patients for now.

**Figure 1:**
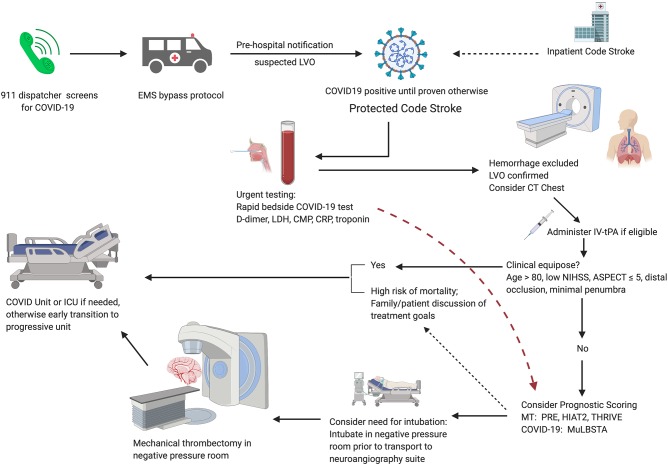
Proposed algorithm for AIS patient triage during the COVID-19 pandemic. Pre-hospital screening for symptoms of COVID-19 should be performed by the 911 dispatcher. EMS implements region-specific bypass protocols for patients with suspected large vessel occlusion. Pre-hospital notification allows for room and team preparation. Immediate lab draws and bedside testing for COVID-19 in CT suite followed by routine stroke imaging; if suspected COVID-19 or unable to obtain history, consider CT chest. If there is clinical equipoise, patients should be managed conservatively. For patients with suspected moderate to severe COVID-19, consider prognostic scoring. Early discussion of treatment goals may be appropriate for patients with severe COVID-19 and a high risk of mortality. For patients selected for MT, consider intubation in a negative pressure room before transferring to the neuroangiography suite.

Although LVO patients with clinical equipoise may be managed conservatively, denial of MT for patients within the established guidelines may lead to poorer clinical outcomes, longer hospital stays, increased need for post-acute care and long-term skilled facility beds, and increased long-term cost of care and resource utilization (56, 66). With appropriate precautions, MT for patients younger than 80 years with LVO and suspected COVID-19 who may potentially benefit is a reasonable approach. In patients younger than 80 years with suspected moderate to severe COVID-19, use of pre-treatment thrombectomy scores such as HIAT2 (67), THRIVE (68), and PRE (69) in conjunction with COVID-19 predictors may provide important insight to guide discussions of treatment goals and expectations of long-term clinical outcome (See Figure 1 for proposed algorithm for patients with suspected LVO).

### Anesthesia and Sedation

Conscious sedation can be performed in patients in whom there is a low risk of intra-procedural conversion to general anesthesia and can maintain sufficient oxygenation without the use of supplemental oxygen. Randomized clinical trials have shown that general anesthesia is safe for patients undergoing MT and should be considered for all MT patients during the COVID-19 pandemic (70, 71). Patients with respiratory failure, need for oxygen supplementation to maintain SpO_2_ ≥ 92% and PaO_2_ > 60 mmHg, or active cough should be intubated in a negative pressure room before transport to the NAS (72, 73). Extension tubing should be set up to avoid circuit disconnections or ventilator transfers once in the NAS. Providers should aim to avoid suctioning during the procedure and non-intubated patients should be provided masks to wear during the procedure.

### Neuroangiography Suite

Negative pressure rooms should ideally have self-closing doors with a negative pressure anteroom to allow for patient transport and an area for donning and doffing. Negative pressure rooms provide a pressure differential between the room and surrounding environment and ensure that the total mechanically supplied air is less than that of the total mechanically exhausted air. This can be achieved by placing a high-efficiency particulate air (HEPA) filter exhausting clean air back to the air system or through a window to the atmosphere and sealing any other exhaust/return air ducts. A pressure monitor should be visible in the room to ensure a pressure differential of −2.5 Pa has been reached. This can be achieved by increasing the flow on the HEPA machine and should provide at least 12 air changes per hour (74). Due to time constraints and architectural challenges in most NAS, it may be reasonable to perform MT in a designated negative pressure COVID swing single plane suite that can be shared with interventional cardiology and/or interventional radiology.

### Mechanical Thrombectomy Procedure and Room Set-Up

Providers should perform all MT with the presumption that patients are COVID-19 positive and wear standard PPE (surgical cap, eye protection, full gown, gloves, shoe covers) and an N95 mask. Prior to MT, all unnecessary equipment should be removed from the NAS and all remaining equipment such as anesthesia machines and other surfaces should be covered with a protective covering. Only equipment and staff necessary for the procedure should be in the room at the onset of the procedure. Supporting staff should be identified as “hot” (exposed to patient) or “cold” (not exposed to patient) prior to the procedure. A “cold” nurse or technician should be available in full PPE in the control room in case additional supplies are needed or if the “hot” technician becomes contaminated. Simulation training with the entire team should be performed to help identify early issues and improve team preparation. If emergent intubation is required during the procedure, all personal should leave the room except those essential for immediate patient care. Signs should be placed on the NAS indicating an ongoing procedure on COVID-19 positive or suspected patient. Current SNIS recommendations include early transition of stroke patients to the step-down unit to minimize the use of ICU beds (56).

When negative pressure rooms are not available, the use of an anteroom is more important, and “foot traffic” near the neuroangiography suite should be minimized. In positive pressure neuroangiography suites, environmental services should wait 207 min or run a HEPA filter in the room for about 20 min before entering the room (75). Terminal cleaning with an EPA registered, hospital-grade disinfectant must be performed between patients. This includes, but is not limited, to disinfecting the floor and walls, all surfaces, especially high touch areas, cables, anesthesia machines, and trash receptacles. The thorough cleaning of the neuroangiography suite often takes about an hour before the room can be turned around.

## Case Study

**Figure 2:**
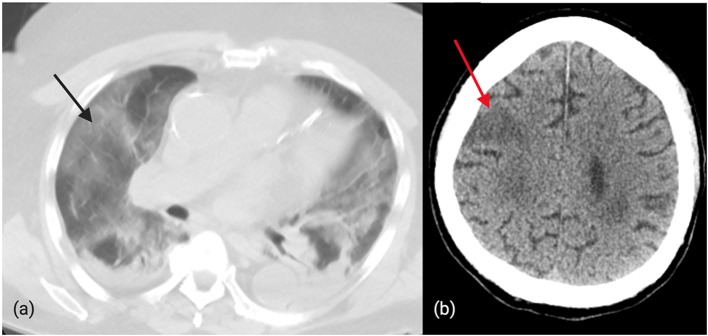
**(a)**: Diffuse, bilateral ground-glass opacities in a patient with confirmed COVID-19 and distal MCA occlusion. **(b)**: CT brain revealing ischemic changes in the right cortical MCA territory in a patient with COVID-19.

Recently, a 75-year-old man with right gaze deviation and left arm drift (initial NIHSS 12) presented to our hospital. The patient was identified by bypass protocols to have a suspected LVO based on the Rapid Arterial oCclusion Evaluation (RACE) score and was brought directly to our comprehensive stroke center. Initial pre-hospital page from emergency medical services (EMS) identified the patient as suspected COVID-19 positive with recent respiratory symptoms as well as “RACE positive.” On arrival, the patient was met by one nurse, CT technician, and physician in PPE. In addition to neuroimaging, a CT chest (Figure 2) was performed which revealed diffuse ground-glass opacities consistent with COVID-19. Vascular imaging revealed a distal right MCA M3 branch occlusion with matching infarct, and labs revealed an elevated troponin (0.26), elevated AST (50), bilirubin (1.3), LDH (523), CRP (27), ferritin (393), and procalcitonin (2.2). Given the lack of clinical-core mismatch, reperfusion therapy was not offered. The patient required intubation and within 2 days, the patient passed away from respiratory failure and shock.

## Conclusion

COVID-19 has placed an unprecedented strain on healthcare systems and resources. Our review summarizes the clinical presentation of COVID-19 and provides prognostic features of severe COVID-19 infection to help determine the risks and benefits of performing MT. While the provided algorithm accounts for many considerations for MT in the current climate, the decision to perform MT is complex and should be individualized. Furthermore, there is a lack of literature on outcomes of MT in LVO patients with COVID-19 and these opinions are based on expert views and anecdotal reports.

Although stroke physicians and neurointerventionalists may not be at the forefront of the COVID-19 battle, we must work to support and protect all other healthcare workers and their families, minimize futile reperfusion, reduce neuroangiography suite turnover, limit PPE use, and ensure that all ventilators and other resources are used for patients who would most likely benefit. In a time when most systems are overwhelmed, canceling elective procedures and minimizing non-urgent diagnostic procedures will free up manpower to take care of COVID-19 patients and reduce healthcare workers' burden. We must continue to advocate for our stroke patients and intervene on patients when we are confident that the risks taken, for the patient as well as the healthcare team are less than the benefits.

## Ethics Statement

Written informed consent was not obtained from the individual(s) for the publication of any potentially identifiable images or data included in this article.

## Author Contributions

HS contributed substantially to the conception and design of the study, drafted and provided critical revision of the article. AC, SZ, RB, and AJ contributed substantially to design of the study and critical revision of the article. MJ contributed substantially to the conception and design of the study, provided critical revision of the article, and provided final approval of the version to publish.

## Conflict of Interest

The authors declare that the research was conducted in the absence of any commercial or financial relationships that could be construed as a potential conflict of interest.
